# A retrospective database study of oral corticosteroid and bisphosphonate prescribing patterns in England

**DOI:** 10.1038/s41533-020-0162-6

**Published:** 2020-02-13

**Authors:** Christos V. Chalitsios, Dominick E. Shaw, Tricia M. McKeever

**Affiliations:** 1 NIHR Division of Respiratory Medicine, University of Nottingham, Nottingham, UK; 2 Division of Epidemiology and Public Health, University of Nottingham, Nottingham, UK

**Keywords:** Preventive medicine, Health services

## Abstract

Exposure to oral corticosteroids (OCS) is associated with an increased risk of osteoporosis and fragility fractures. Guidelines suggest bisphosphonate (BP) therapy as the first-line treatment of glucocorticoid-induced osteoporosis (GIOP). This population study used publicly available data, including prescription annual cost analysis and monthly practice-level data. Our aim was to examine the prescribing of OCS and BP at practice level and investigate reasons for variation using a mixed-effect negative binomial regression analysis. There was a rise in OCS and BP prescriptions of 55% and 1200% from 1998 to 2018, respectively. Of the 6586 included practices, the median (IQR) of OCS and BP prescriptions were 120.8 (84.8–160.4) and 107.7 (73.8–147.4) per 1000 patients, respectively. Asthma and chronic obstructive pulmonary disease (COPD) were significantly associated with OCS use (*p* < 0.0001), but only COPD was associated with BP use (*p* < 0.0001). Higher OCS prescribing rates were associated with higher BP prescribing rates (5th to 1st quintile—IRR = 1.99; 95% CI: 1.88–2.10). Practice list size, deprivation and advanced age were all associated with both drugs (*p* < 0.0001). In conclusion, although OCS use is positively associated with BP prescription, variation among practices and CCGs exists. The variation in prescribing suggests there is still a need to improve GIOP prevention.

## Introduction

Oral corticosteroids (OCS) (glucocorticoids) are used to treat chronic conditions including autoimmune,^1^ and respiratory diseases.^2,3^ Asthma and COPD are two of the most common indications for prolonged OCS use (more than 90 days).^4^ Both short-term (5−90 days) and prolonged exposure to OCS can lead to deleterious effects^5,6^ including bone loss resulting in osteoporosis and fragility fracture.^7^ Bone loss is substantial and rapid during the first months of the OCS treatment.^8^ Patients with severe asthma exposed to prednisolone 5 mg per day are more likely to be diagnosed with osteoporosis (OR = 6.5) and have a fracture (OR = 1.5) compared to those without asthma.^9^ After OCS initiation, spine fracture risk increases by 55% with exposure at doses as low as prednisone 2.5 mg per day, whereas hip fracture risk goes up by 50% among patients exposed to 2.5–7.5 mg per day.^10,11^

Fragility fractures are also associated with substantially increased healthcare costs, morbidity, and mortality,^12,13^ and guidelines suggest all patients exposed to any dose of OCS for more than 3 months should be considered for BP therapy to prevent glucocorticoid-induced osteoporosis (GIOP).^14,15^ The bisphosphonate class is effective in reducing bone loss and fragility fracture risk.^16,17^ Despite this, only a minority of patients with increased fragility fracture risk receive appropriate therapy.^18,19^ There are no specific guidelines for GIOP in asthma or COPD and the size of the potential problem is not well established.

We are unaware of any published U.K. research investigating the trends in OCS and anti-GIOP therapy (BP) prescribing. Our aim was to comprehensively assess OCS and BP prescribing patterns, at practice level, using primary care data from England and to investigate factors associated with their prescribing, in order to gain a better understanding of prescribing enabling us to reduce prescribing variation and optimise GIOP prevention.

## Results

### Practice characteristics

In our analysis, we included 195 Clinical Commissioning Groups (CCGs) containing 6586 practices after the exclusion of 507. In 2018, the median (IQR) OCS and BP prescriptions per 1000 patients was 120.8 (84.8–160.4) and 107.7 (73.8–147.4), respectively. The characteristics of practices are summarised in Table 1.

**Table 1:** Characteristics of practices included in the analysis (from January 2018 to December 2018).

	Median	IQR
Asthma prevalence (%)	6.0	5.1–6.8
COPD prevalence (%)	1.8	1.3–2.5
Practice list size	7478	4664–11,270
Patients with long-term health conditions (%)	51.7	45.5–59.1
Patients over 65 years old (%)	17.4	12.2–21.8
Quality Outcomes Framework score	549.0	534.7–556.8
OCS prescribed items per 1000 patients	120.8	84.8–160.4
BP prescribed items per 1000 patients	107.7	73.8–147.4

*COPD* chronic obstructive pulmonary disease, *OCS* oral corticosteroids, *BP* bisphosphonates.

### Long-term patterns and ratio between OCS and BP prescriptions

**Fig. 1:**
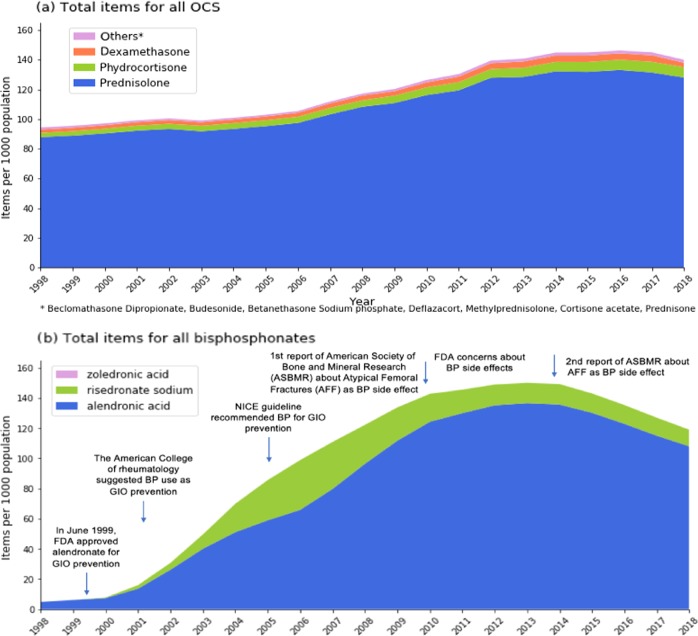
Long-term prescribing patterns. Total (**a**) oral corticosteroids (OCS) and (**b**) bisphosphonates (BP) prescribed items per 1000 population over the period from 1998 to 2018. The arrows provide factors that may have affected their prescribing.

Prednisolone was the most frequently prescribed OCS. There was a steady increase in OCS prescriptions over time (Fig. 1a). In 1998, there were 95 OCS prescriptions per 1000 population increasing to 140 in 2018 (55% rise). The cost of OCS was £250 per 1000 population until 2006, with a noticeable increase up to just less than £2000 the following years (Supplementary Fig. 1).

There was an increase in bisphosphonate prescribing rates over time (Fig. 1b). In 1998, there were 10 BP prescriptions per 1000 population, while the total prescriptions reached 120 in 2018 (1200% increase). The most prescribed bisphosphonate was alendronic acid. BP cost peaked at £3200 per 1000 population in 2005; however, there was a reduction to £101 by 2018 (Supplementary Fig. 1).

There were 0.99 OCS prescriptions per 1 BP item in 2015; however, this relationship changed slightly to 1.16 by 2018 (Table 2).

**Table 2:** Trends in ratio between oral corticosteroids and bisphosphonates over the period of 2015−2018.

Year	OCS items	BP items	Ratio scale (OCS/BP)
2015	7,781,584	7,836,568	0.99
2016	7,958,014	7,479,733	1.06
2017	7,911,005	7,062,931	1.12
2018	7,799,798	6,728,997	1.16

*OCS* oral corticosteroids, *BP* bisphosphonates.

### Variations among practices and CCGs for OCS and BP items

**Fig. 2:**
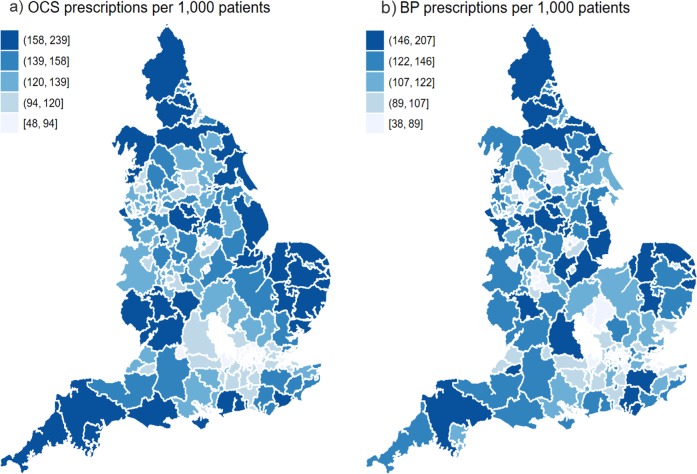
Geographical variation in prescribing. Geographical variations in (**a**) oral corticosteroids (OCS) and (**b**) bisphosphonates (BP) prescribed items categorised into quantiles among Clinical Commissioning Groups from January to December 2018.

In 2018, there was a significant variation between OCS (*m* = 129.6, SD = 38.9) and BP (*m* = 118.5, SD = 34.2) prescription per 1000 patients; *t* = 6.27, *p* < 0.0001. OCS prescription varied between 48 and 239 and BP ranged from 38 to 207 prescriptions per 1000 patients across CCGs. Sixty out of 195 CCGs prescribed less OCS than BP items, and 135 more OCS than BP items per 1000 patients (Fig. 2).

### Factors associated with OCS and BP prescribing

We found that OCS prescriptions were associated with the factors listed in Table 3 apart from the QOF score and percentage of patients with a long-term health disease. Asthma and COPD were significantly associated with the OCS use (*p* < 0.0001). The percentage of patients aged 65 years old or more was the strongest predictor of OCS prescriptions (*p* < 0.0001). Practices in the highest quintile prescribed 1.74 times more OCS (IRR = 1.74; 95% CI: 1.64–1.84) than those in the lowest one. Practise list size was also a positive predictor of OCS prescribing (*p* < 0.0001) and the most deprived areas were less likely to prescribe less OCS than the least deprived areas (IRR = 0.84; 95% CI: 0.80–0.88).

**Table 3:** Oral corticosteroids prescribing rates in 2018, stratified by seven practice characteristics and two respiratory diseases in a negative binomial model reporting incidence rate ratio.

	Quintile range	Median OCS prescription per 1000 patients	Univariate model IRR (95% CI)^a^	Multivariate model IRR (95% CI)^b^	*p* value^c^
Asthma prevalence (%)	≤4.85	69.26	Reference	Reference	<0.0001
	4.86–5.67	109.48	1.46 (1.39–1.51)	1.11 (1.08–1.15)	
	5.68–6.29	128.56	1.69 (1.61–1.76)	1.18 (1.13–1.23)	
	6.30–6.98	144.31	1.90 (1.82–1.98)	1.23 (1.18–1.29)	
	6.99–12.56	157.61	2.10 (2.01–2.19)	1.28 (1.22–1.34)	
COPD prevalence (%)	≤1.17	69.68	Reference	Reference	<0.0001
	1.18–1.65	107.95	1.48 (1.42–1.55)	1.13 (1.09–1.17)	
	1.66–2.10	128.56	1.77 (1.70–1.85)	1.20 (1.15–1.25)	
	2.11–2.69	144.67	1.95 (1.87–2.04)	1.26 (1.21–1.33)	
	2.70–10.45	155.17	2.10 (2.01–2.20)	1.34 (1.27–1.41)	
Practice list size	≤4,127	112.14	Reference	Reference	<0.0001
	4128–6287	120.98	1.03 (0.98–1.08)	1.01 (0.97–1.05)	
	6290–8809	127.64	1.04 (0.99–1.09)	1.00 (0.97–1.05)	
	8810–12,335	129.77	1.05 (1.01–1.10)	0.98 (0.95–1.02)	
	≥12,336	118.48	0.91 (0.86–0.95)	0.88 (0.85–0.91)	
QOF score	≤529.81	111.24	Reference	Reference	0.2579
	529.82–545.04	115.02	1.05 (1.01–1.11)	1.03 (0.99–1.07)	
	545.05–552.16	118.01	1.08 (1.03–1.13)	1.02 (0.99–1.06)	
	552.17–557.84	128.73	1.15 (1.10–1.21)	1.01 (0.98–1.06)	
	557.85–559	137.49	1.24 (1.18–1.30)	1.02 (0.98–1.07)	
% over 65 years old	≤11.00	68.16	Reference	Reference	<0.0001
	11.01–15.63	106.01	1.50 (1.44–1.56)	1.24 (1.20–1.28)	
	15.64–18.86	126.68	1.78 (1.70–1.85)	1.39 (1.34–1.46)	
	18.87–22.77	139.56	1.95 (1.87–2.04)	1.49 (1.42–1.57)	
	≥22.78	172.23	2.40 (2.30–2.50)	1.74 (1.64–1.84)	
% patients with a long-term health disease	≤43.95	73.53	Reference	Reference	0.1923
	43.97–49.65	109.62	1.40 (1.34–1.47)	1.03 (0.99–1.06)	
	49.66–53.85	128.86	1.64 (1.56–1.71)	1.04 (1.01–1.08)	
	53.86–58.44	140.36	1.77 (1.69–1.85)	1.03 (0.99–1.07)	
	≥58.45	151.76	1.91 (1.83–2.00)	1.04 (0.99–1.08)	
IMD score	Least deprived	126.98	Reference	Reference	<0.0001
	—	134.29	1.03 (0.98–1.08)	0.92 (0.88–0.95)	
	—	122.00	0.96 (0.91–1.01)	0.87 (0.83–0.90)	
	—	115.85	0.89 (0.85–0.94)	0.85 (0.81–0.88)	
	Most deprived	110.01	0.88 (0.84–0.92)	0.84 (0.80–0.88)	

*OCS* oral corticosteroids, *COPD* chronic obstructive pulmonary disease, *QOF* Quality and Outcomes Framework, *IMD* Index of Multiple Deprivation.

^a^Negative binomial model.

^b^Mixed-effects negative binomial model.

^c^From multivariate analysis using the likelihood ratio test.

OCS were associated with BP prescriptions (*p* < 0.0001), with higher OCS prescribing rates to have been associated with higher BP prescribing rates (5th to 1st quintile—IRR = 1.99; 95% CI: 1.88–2.10) (Table 4). COPD was significantly associated with a BP prescription (*p* < 0.0001), but asthma was not (*p* = 0.6848). Practices located in more deprived areas were 28% less likely to prescribe less BP than the least deprived practices (IRR = 0.72; 95% CI: 0.68–0.77).

**Table 4:** Bisphosphonates prescribing rates in 2018, stratified by seven practice factors and OCS per 1000 patients in a negative binomial model reporting incidence rate ratio.

	Quintile range	Median BP prescription per 1000 patients	Univariate model IRR (95% CI)^a^	Multivariate model IRR (95% CI)^b^	*p* value^c^
Asthma prevalence (%)	≤4.85	72.87	Reference	Reference	0.6848
	4.86–5.67	100.36	1.23 (1.21–1.33)	1.01 (0.96–1.04)	
	5.68–6.29	115.37	1.44 (1.38–1.52)	1.04 (0.99–1.08)	
	6.30–6.98	121.57	1.55 (1.47–1.63)	1.01 (0.96–1.05)	
	6.99–12.56	128.56	1.67 (1.59–1.76)	1.01 (0.97–1.07)	
COPD prevalence (%)	≤1.17	67.37	Reference	Reference	<0.0001
	1.18–1.65	98.71	1.40 (1.37–1.47)	1.08 (1.04–1.12)	
	1.66–2.10	115.18	1.63 (1.56–1.72)	1.13 (1.08–1.19)	
	2.11–2.69	123.80	1.72 (1.64–1.80)	1.15 (1.09–1.21)	
	2.70–10.45	131.90	1.85 (1.76–1.94)	1.19 (1.12–1.26)	
OCS per 1000 patients	≤75.37	52.66	Reference	Reference	<0.0001
	75.40–108.44	89.60	1.63 (1.56–1.70)	1.39 (1.34–1.46)	
	108.56–136.24	110.94	2.00 (1.91–2.09)	1.58 (1.51–1.66)	
	136.27–169.84	128.78	2.32 (2.22–2.43)	1.74 (1.66–1.83)	
	≥169.85	161.98	2.94 (2.82–3.07)	1.99 (1.88–2.10)	
Practice list size	≤4127	102.46	Reference	Reference	<0.0001
	4128–6287	110.85	1.03 (0.98–1.09)	0.99 (0.96–1.03)	
	6290–8809	113.50	1.04 (0.98–1.08)	0.97 (0.93–1.01)	
	8810–12,335	112.61	1.04 (0.98–1.09)	0.97 (0.90–0.97)	
	≥12,336	104.82	0.90 (0.85–0.95)	0.96 (0.90–0.91)	
QOF score	≤529.81	97.30	Reference	Reference	<0.0001
	529.82–545.04	102.15	1.04 (0.99–1.10)	1.05 (1.01–1.09)	
	545.05–552.16	106.40	1.08 (1.03–1.14)	1.07 (1.03–1.11)	
	552.17–557.84	115.61	1.16 (1.10–1.23)	1.07 (1.03–1.11)	
	557.85–559	123.05	1.25 (1.19–1.32)	1.09 (1.04–1.13)	
% over 65 years old	≤11	54.77	Reference	Reference	<0.0001
	11.01–15.63	95.43	1.63 (1.56–1.71)	1.35 (1.29–1.40)	
	15.64–18.86	112.63	1.98 (1.89–2.07)	1.50 (1.42–1.58)	
	18.87–22.77	127.56	2.20 (2.10–2.30)	1.59 (1.50–1.68)	
	≥22.78	154.39	2.66 (2.54–2.78)	1.82 (1.71–1.94)	
% patients with a long-term health disease	≤43.95	70.30	Reference	Reference	0.3457
	43.97–49.65	98.73	1.36 (1.30–1.43)	1.03 (0.99–1.08)	
	49.66–53.85	116.34	1.56 (1.49–1.64)	1.03 (0.98–1.08)	
	53.86–58.44	123.56	1.66 (1.59–1.75)	1.01 (0.96–1.05)	
	≥58.45	131.17	1.82 (1.73–1.91)	1.04 (0.99–1.09)	
IMD score	Least deprived	117.83	Reference	Reference	<0.0001
	—	122.89	1.03 (0.97–1.18)	0.93 (0.90–0.97)	
	—	111.17	0.92 (0.87–0.96)	0.85 (0.82–0.90)	
	—	102.84	0.84 (0.81–0.88)	0.80 (0.76–0.84)	
	Most deprived	85.29	0.74 (0.70–0.78)	0.72 (0.68–0.77)	

*BP* bisphosphonates, *COPD* chronic obstructive pulmonary disease, *OCS* oral corticosteroids, *QOF* Quality and Outcomes Framework, *IMD* Index of Multiple Deprivation.

^a^Negative binomial model.

^b^Mixed-effects negative binomial model.

^c^From multivariate analysis using the likelihood ratio test.

The CCG to which a practice belongs was significantly associated with prescribing rates and accounted for 11% and 5% of the variation in OCS and BP prescribing, respectively.

We also found similar results in the other years (Supplementary Tables 4–12).

## Discussion

Overall, we observed an increase in prescribed OCS and BP items between 1998 and 2018. We found large variation in prescribing rates across practices in England. Asthma and COPD were significantly related with OCS prescriptions, but only COPD with BP. Patient list size, deprivation and advanced age were all associated with both the drugs. The CCG to which each practice belongs also contributed to the prescribing variation. Finally, OCS was positively associated with BP prescriptions.

The increase in BP prescriptions (2000–2010) could be explained by the uptake of clinical guidance. The FDA approved alendronate use in June 1999^20^; the first guidance for the GIOP prevention from the American College of Rheumatology was published in 2001^21^ and NICE recommended BP as first-line GIOP prophylaxis in 2005.^22^ The plateauing of BP prescriptions from 2010 with a downward trend from 2015, in contrast with the steady OCS prescriptions, may reflect the FDA concerns^23,24^ about BP side effects such as osteonecrosis of the jaw and atypical femoral fractures. In 2014, the release of the second report of American Society of Bone and Mineral Research provided a more robust evidence about the atypical fractures as BP side effects probably was the reason for a further decrease.^25^ An investigation in the USA found a similar 50% reduction in BP use between 2008 and 2012 following concerns about their safety.^26^ However, these side effects might be greater concern in younger age groups, as they will benefit less using BP. Our findings are also consistent with two other studies that found a steady increase in BP use from 2000 onwards.^27,28^

Establishing an optimal ratio of BP to OCS prescription is challenging. Τhe management of multi-morbidities makes prescribing decisions complex and although prescriptions should be guideline informed, they should not be guideline directed.^29,30^ Initiation of BP medication may also depend on how long past 3 months OCS exposure is expected. There is good evidence that the benefits of BP in preventing GIOP outweigh the risks^25,31,32^ and bone mineral density testing is recommended within 6 months after OCS initiation, repeating it every 1–3 years.^33^ Other potential options for GIOP may be recommended (Vitamin D, HRT), but have less data.

Our analysis demonstrates geographical variation across practices and CCGs in prescribing rates of both classes of drugs and in between the drugs. Apart from variation associated with practice factors, our results revealed that CCGs accounted for variation in each medication that may indicate differences in prescribing policy. Interestingly, prescribing differences related to deprivation, in terms of OCS, may reflect inequity of access to treatment difference, whereas in BP rates, may reflect access to DEXA scanning. Our findings are consistent with another UK study that found marked regional differences in the anti-osteoporosis prescribing rates.^26^ Other studies have also confirmed the impact of deprivation and the other examined factors on variation in drug prescribing.^34–36^

Despite the proven benefits of BP as an osteoporosis therapy, there is evidence that they are underutilised both in the UK and USA.^19,28^ Addressing this issue will hinge on education in both primary and secondary care, and provision of suitable guidelines. One practical solution in healthcare systems that use electronic records/prescribing would be to flag patients who meet BP criteria based on age, gender and OCS use. Alerts already occur for a number of conditions (including excess salbutamol use) and this flag could be incorporated into the chronic disease review.

Although there is clear guidance on OCS and BP therapy, there is no current recommendation for BP therapy for inhaled corticosteroid (ICS) users, despite evidence supporting an increased osteoporotic fracture risk related to ICS.^37–39^ It is best practice to review ICS dose and use the lowest dose possible to maintain asthma control.^40^

We also found that the rise in OCS cost was mainly driven by a steep increase in price of hydrocortisone tablets from 2008 onwards; and BP prescribing went up as BP cost went down after 2005.

To our knowledge, this is the first study to examine the OCS and BP prescribing patterns and their association with practice-level factors. We use real prospectively collected prescribing data based on NHS Digital files and included practices and CCGs covering the entire country. There are some limitations; we could not evaluate prescriptions in secondary care. Secondly, we were not able to know the indication for each prescription. Thirdly, we could not perform individual-level analysis in order to identify the OCS prescriptions that need BP therapy according to the guidelines’ recommendations.

In conclusion, the overall levels of OCS and BP prescription have increased since 1998. Concerns about BP adverse effects may account for a latter reduction in BP prescriptions in contrast to steady or increased OCS prescriptions. We found clear variation in OCS and BP, and this unwarranted variation appears to be driven to a large extent by factors including deprivation, patient list size and CCGs. The variation in prescribing suggests there is still a need to improve GIOP prevention.

## Methods

### Data sources

We used national data based on the annual prescription cost analysis (PCA) from 1998 to 2018, and monthly practice-level data files by NHS Digital from January 2015 to December 2018.

The annual prescription cost analysis data files provide details of the number of items and net ingredient cost of all prescriptions for each medication dispensed in the community in England. Data were normalised by converting to relative figures per 1000 population using mid-year population estimate for England.^41^ The monthly data published by NHS Digital consist of one row for each medication providing information about the prescribing volume and cost for each practice in England. These data are based on claims from community pharmacies and contains items that have been dispensed.

We extracted the data from OpenPrescribing (https://openprescribing.net/) database. OpenPrescribing is built by EBM Data Lab and provides a search interface onto the raw prescribing data files published by NHS, making the access to this complex information easier in order for these data to be more impactful in real world. There are approximately 900 million rows of these data. New data are released every month; however, they are 2 months behind (i.e. March’s prescribing data are published in May). OpenPrescribing has data from the past 5 years without containing any patient information and indication of the treatment length.

### Study design

We conducted a retrospective cohort with ecological elements study on all English practices and CCGs, measuring patterns in OCS and BP prescribing items over time at practice level. We described the ratio in prescribing rate between OCS and BP and measured any variation among CCGs geographically. We also matched the monthly practice-level data with open access data derived from Public Health England to investigate for reason for any variation in OCS and BP prescribing at practice and CCG level.

### Drugs extraction

We used prescribed “items” as a measure of prescribing. A prescription item refers to a single supply of a medicine prescribed on a prescription form. If a prescription form includes three medicines, it is counted as three prescription items.

We extracted prescribing and cost data for the following OCS as are stated on OpenPrescribing.net: Beclometasone Dipropionate (Systemic) (Brand name: Clipper), Budesonide (Brand names: Entocort, Budenofalk) derived from section “1.2.5: Corticosteroids” of the British National Formulary (BNF) book. Betamethasone Sodium Phosphate (brand names: Betnesol, Betameth sod phos, all systemic), Cortisone Acetate (brand name: Cortisone acet), Deflazacort (brand name: Calcort), Dexamethasone (brand name: Dexameth (systemic)), Hydrocortisone (brand name: Hydrocortone, Hydrocort (systemic)), Methylprednisolone (brand name: Medrone (systemic)), Prednisolone (brand name: Prednisolone (systemic)), and Prednisone (brand name: Lodotra) were derived from section “6.3.2: Glucocorticoid therapy” of the BNF (Supplementary Table 2).

We extracted data about bisphosphonates following guidance from the National Osteoporosis Guideline Group.^12^ These were: Alendronic acid (brand names: Alendronic acid, Fosamax), risedronate sodium (brand names: Actonel, Risedronate sod) and Zoledronic acid (brand name: Zometa). All BP were checked against section “6.6.2: Bisphosphonates and other drugs” of the BNF (Supplementary Table 1).

### Long-term patterns

Data from OpenPrescribing.net based on PCA were obtained describing the annual patterns in OCS and BP prescribing items and cost per 1000 population from 1998 to 2018. We aggregated the total annual items/cost per 1000 population of each OCS/BP type. We created stacked graphs to depict the annual volume of each chemical of each drug per 1000 population.

### Ratio between OCS and BP prescriptions

We extracted monthly data about OCS and BP prescribed items per CCG from January 2015 to December 2018. We aggregated the monthly items of each CCG per year obtaining the total annual number of both OCS and BP items. The ratio between the above classes of drugs was calculated using:

$${\mathrm {Ratio}} = \left( {\frac{{{\mathrm {Total}}\,{\mathrm {OCS}}\,{\mathrm {items}}\,{\mathrm {the}}\,{\mathrm {year}}\,{\mathrm {of}}\,{\mathrm {interest}}}}{{{\mathrm {Total}}\,{\mathrm {BP}}\,{\mathrm {items}}\,{\mathrm {the}}\,{\mathrm {year}}\,{\mathrm {of}}\,{\mathrm {interest}}}}} \right).$$

### Variations among CCGs for OCS and BP items

In 2012, each practice was grouped into CCGs. The CCGs are responsible for the planning and commissioning of healthcare in a local community. To examine geographical variations in OCS and BP prescribing in 2018, practices were grouped by CCG. The prescribing rate per 1000 patients of dispensed OCS/BP for each CCG was derived by dividing the total number of prescribing items by the mean patient list size over the year in each CCG, multiplied by 1000. Then, they were categorised into quantiles.

### Associations between OCS and BP prescribing

After calculating the rate of OCS and BP items per 1000 patients per practice, we determined other independent variables to examine which indicators were associated with OCS and BP prescription in 2018. We repeated the analysis for 2015, 2016 and 2017.

Data were extracted on the following factors from the Public Health England (https://fingertips.phe.org.uk/profile/general-practice/data): the percentage of (a) asthma diagnosis, (b) COPD diagnosis, (c) patients over 65 years old, (d) patients with a long-term health condition defined as the percentage of people who answered “Yes” in the practices’ patient survey (http://www.gp-patient.co.uk/practices-search) question: “Do you have any long-term physical or mental health conditions, disabilities or illnesses?”, (e) the Index of Multiple Deprivation (IMD) score, and (f) the mean practice list size.

All of the above figures were derived from the correspondence year of interest apart from the IMD score which was available only for 2015; we used this score for the analysis in each year of interest. All of the above indicators were obtainable based on the CCGs which were active in 2017/2018. Furthermore, we extracted data about the Quality and Outcomes Framework Score (QOF) score (https://digital.nhs.uk/data-and-information/publications/statistical/quality-and-outcomes-framework-achievement-prevalence-and-exceptions-data) by practice. We used the 2017/2018 QOF score not only in the analysis for the year 2017 but also in 2018 as the 2018/2019 QOF score had not been released yet. Additionally, we excluded practices without a QOF or having a list size less than 1000 patients (Supplementary information, Practice exclusion).

### Statistical methods

Practices’ characteristics as well as OCS and BP prescriptions were analysed using descriptive analysis reporting them as median along with IQR. A paired *t* test was performed examining the significance of variation between OCS and BP prescription among CCGs. To examine the association between OCS/BP per 1000 patients and the factors, we conducted a negative binomial regression analysis. We stratified the rate of OCS and BP prescribing for each one of the investigating factors. We put these factors in the model. We also split the OCS per 1000 patients into quintiles and put them in the BP analysis. Afterwards, as healthcare policies and commissioning differ among CCGs, we used a mixed-effect negative binomial model, defining the rate of OCS and BP prescriptions per 1000 patients as the dependent variable. We used the factors defined above as fixed-effects explanatory variables, and the CCG of each practice as a random-effect variable. The variables were grouped into quintiles to allow for nonlinearity of effects. IRRs with a 95% CI were used to determine the strength of associations and *R*^2^ to show the value and significance of variance associated with CCG grouping. *P* values < 0.05 were considered statistically significant. Practices with missing values were less than 0.25%.

Python was used for data management. Both graphs and maps were constructed with Python. Statistical analysis was conducted by using Stata, version 16.

### Reporting summary

Further information on experimental design is available in the Nature Research Reporting Summary linked to this article.

## Supplementary information

Reporting Summary

Supplementary Information

## Data Availability

The datasets generated and/or analysed during the current study are available from the corresponding author on reasonable request. The datasets can be found on 10.6084/m9.figshare.11590707.

## Code availability

The program codes used during the current study are available from the corresponding author on reasonable request.
