# Supplementary material for: A retrospective database study of oral corticosteroid and bisphosphonate prescribing patterns in England
Source: NPJ Prim Care Respir Med. 2020 Feb 13;30:5. doi: 10.1038/s41533-020-0162-6 (PMC7018734; doi:10.1038/s41533-020-0162-6)
Supplement: Supplementary file 2 — Supplementary Information [file 41533_2020_162_MOESM2_ESM.pdf]

Supplementary information

A retrospective database study of oral corticosteroid and bisphosphonate prescribing patterns in England

Christos V. Chalitsios[1, *], Dominick E. Shaw[1], Tricia M. McKeever[2]

[1] NIHR Division of Respiratory Medicine, University of Nottingham, Nottingham, UK

[2] Division of Epidemiology and Public Health, University of Nottingham, Nottingham, UK

Practice exclusion

In 2018, there were 7,093 practices from 195 CCGs. Initially, all practices (n=255) without having achieved a QOF score have been excluded and then, all practices (n=331) with a patient list size less than 1,000 patients. Firstly, practices without a having achieved a QOF score may have simply opted out and could be normal active practices, but there is likely to be a high proportion of unusual practices amongst those without scores. For example, practices may opt out because they are in the process of opening, closing or merging, be under special measures or other temporary closure, have very few patients in the relevant clinical areas, or perhaps are very small and don't have the resource or incentive to complete the necessary paperwork. Secondly, practices with a small patient list size were serving a population which is sufficiently different “atypical” (e.g. serving elderly or homeless populations) (1). We used a limit of 1,000 patients throughout our analysis because this has been used elsewhere (2,3)

Furthermore, it was not possible to compare the practices without a QOF score with the included ones, as the majority of them had missing values in all variables (Table 1). However, this can confirm the fact that the excluded practices were inactive through the whole year or for a long period during this.

Supplementary Table 1. Number of practices with missing data per variable.

	Missing data
Variables	Practices without a QOF score (n=255)
Long term health conditions	182
IMD	182
≥65 y.o	184
COPD (%)	251
Asthma (%)	251
QOF	255

References

1. NHS England / Primary Care Commissioning. *Guidance Note: GP Practices serving Atypical Populations.* 2016. https://www.england.nhs.uk/wp-content/uploads/2016/12/atypical-commissioning-guid.pdf

2. Walker J. et al. Trends, geographic variation, and factors associated with prescribing of gluten-free foods in English primary care: a cross sectional study. *BMJ Open* **8**, e021312 (2018)

3. Curtis, H.J et al*.* Time trends and geographical variation in prescribing of antibiotics in England 1998–2017. *J Antimicrob Chemother* **74**, 242–250 (2019)

Drugs codes which were used for the data extraction from OpenPrescribing

Supplementary Table 2. Codes of each chemical substance for the data extraction from OpenPrescribing

Chemical substances	Codes
**Beclometasone Dipropionate**	0105020G0
**Budesonide**	0105020A0
**Betamethasone Sodium Phosphate**	0603020C0
**Cortisone Acetate**	0603020F0
**Deflazacort**	0603020I0
**Dexamethasone**	0603020G0
**Hydrocortisone**	0603020J0
**Methylprednisolone**	0603020S0
**Prednisolone**	0603020T0
**Prednisone**	0603020X0
**Alendronic Acid**	0606020A0
**Zoledronic Acid**	0606020V0
**Ibandonic Acid**	0606020W0
**Risedronate sodium**	0606020R0

Cost of medications

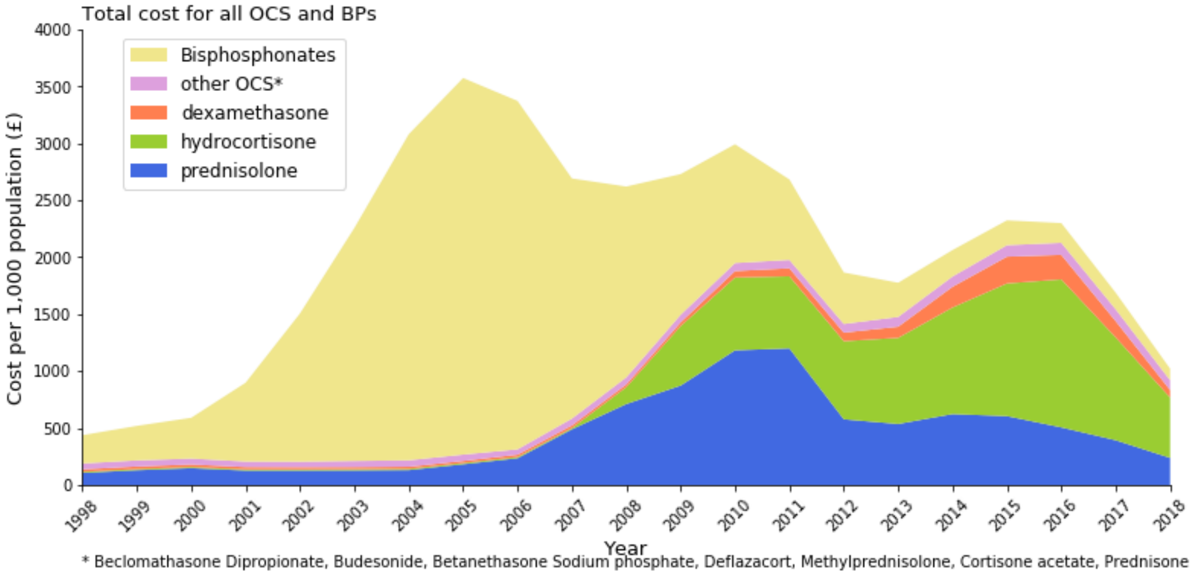

Supplementary Figure 1. Total cost of oral corticosteroids and bisphosphonates prescribed items per 1,000 population over the period from 1998 to 2018.

Variations among practices for OCS and BP items

We demonstrated the annual trends from 2015 to 2018 by calculating deciles at practice-level.

In practices, the 5th decile of OCS and BP prescription declined from 128.8 to 120.8 and 134.8 to 107.7 per 1,000 patients, respectively between 2015 and 2018. The 10th decile remained around the same at 237 for OCS prescription, however the BP ones decreased to 206.3 per 1,000 patients throughout this period (Supplementary Table 3).

Supplementary Table 3. Annual practice deciles of oral corticosteroids and bisphosphonates prescriptions per 1,000 patients (2015 to 2018).

Decile/	OCS prescribed items				BP prescribed items			
Year	2015	2016	2017	2018	2015	2016	2017	2018
1st	60.0	61.1	60.3	48.9	56.5	53.4	50.6	36.4
2nd	82.0	82.1	80.7	72.6	81.3	77.1	72.7	62.4
3rd	99.4	100.2	99.1	91.6	101.0	95.7	88.7	79.9
4th	111.9	115.9	113.7	107.3	117.9	111.4	104.0	94.1
5th	128.8	130.5	128.5	120.8	134.8	126.5	118.3	107.7
6th	148.6	149.2	143.5	135.4	150.1	144.0	132.9	117.5
7th	164.8	167.7	162.8	155.8	169.9	162.5	144.9	131.3
8th	190.2	194.5	185.0	174.3	192.4	189.9	167.2	151.8
9th	222.4	234.6	222.6	203.3	232.2	231.9	200.8	185.0
10th	236.7	259.3	244.1	238.8	249.7	243.7	218.4	206.3

BP, Bisphosphonates; OCS, Oral Corticosteroids.

Factors associated with OCS and BP prescribing

In 2017

Supplementary Table 4. Practice summary characteristics in 2017

	Median	IQR
**Asthma prevalence (%)**	6.0	5.1 - 6.8
**COPD prevalence (%)**	1.8	1.3 - 2.4
**GP list size**	7,273	4,462 - 10,885
**Patients with long-term health conditions (%)**	53.8	48 - 59
**Patients over 65 years old (%)**	17.2	12 - 22
**Quality Outcomes Framework score**	549	535 - 557
**OCS prescribed items per 1,000 patients**	128.5	86.9 - 163.4
**BP prescribed items per 1,000 patients**	118.3	76.9 - 154.6

*From January to December
COPD, Chronic Obstructive Pulmonary Disease; GP, General Practice; OCS, Oral Corticosteroids; BP, Bisphosphonates.

Supplementary Table 5. Oral corticosteroids prescribing rates in 2017 stratified by five GP characteristics factors and two respiratory diseases in a negative binomial model reporting incidence rate ratio

	Quintile range	Median OCS prescription per 1,000 patients	Univariate model IRR (95%CI)*	Multivariate model IRR (95%CI)$	*p*-value**, ^
**Asthma Prevalence (%)**	≤4.84	73.83	Reference	Reference	<.0001
	4.85-5.65	112.40	1.45 (1.39-1.52)	1.13 (1.08-1.16)	
	5.66-6.29	128.20	1.64 (1.57-1.71)	1.18 (1.13-1.23)	
	6.30-6.96	147.06	1.86 (1.78-1.94)	1.21 (1.16-1.26)	
	6.97-14.37	160.38	2.03 (1.95-2.12)	1.28 (1.22-1.32)	
**COPD Prevalence (%)**	≤1.14	72.06	Reference	Reference	<.0001
	1.15- 1.60	110.58	1.45 (1.39-1.52)	1.11 (1.07-1.15)	
	1.61-2.04	132.96	1.73 (1.65-1.80)	1.19 (1.14-1.24)	
	2.05-2.64	148.27	1.92 (1.84-2.01)	1.27 (1.21-1.33)	
	2.65-11.25	158.29	2.04 (1.96-2.13)	1.34 (1.27-1.41)	
**GP list size**	≤3,947	112.46	Reference	Reference	<.0001
	3,949-6,050	122.46	1.03 (0.98-1.08)	1.01 (0.97-1.02)	
	6,051-8,508	129.68	1.06 (1.01-1.11)	1.01 (0.98-1.02)	
	8,509-11,920	134.14	1.07 (1.02-1.13)	0.99 (0.95-1.06)	
	≥11,922	124.35	0.95 (0.90-0.99)	0.90 (0.86-1.16)	
**QOF score**	≤530.81	108.84	Reference	Reference	.0589
	530.82-545.04	115.04	1.08 (1.03-1.13)	1.06 (1.01-1.10)	
	545.05-553.16	123.93	1.14 (1.09-1.19)	1.04 (1.01-1.08)	
	553.17-557.84	133.85	1.20 (1.14-1.26)	1.04 (1.01-1.05)	
	557.85-559	141.05	1.29 (1.23-1.35)	1.05 (1.01-1.09)	
**% over 65 years old**	≤11	70.17	Reference	Reference	<.0001
	11.01-15.63	108.04	1.48 (1.42-1.55)	1.24 (1.17-1.27)	
	15.64-18.86	130.58	1.72 (1.65-1.80)	1.33 (1.27-1.39)	
	18.87-22.43	144.23	1.92 (1.84-2.00)	1.43 (1.36-1.51)	
	≥22.44	173.34	2.30 (2.21-2.40)	1.63 (1.54-1.72)	
**% patients with a long-term health disease**	≤47.28	88.92	Reference	Reference	.0388
	47.29-52.13	114.96	1.28 (1.23-1.33)	1.05 (1.01-1.09)	
	52.14-55.50	129.53	1.47 (1.40-1.54)	1.04 (1.01-1.08)	

	55.51-60.03	143.10	1.56 (1.50-1.64)	1.04 (1.01-1.19)	
	≥60.04	152.62	1.71 (1.63-1.79)	1.06 (1.01-1.10)	
**IMD score**	Least deprived	129.11	Reference	Reference	<.0001
	-	138.69	1.04 (1.00-1.09)	0.94 (0.90-0.97)	
	-	125.62	0.96 (0.92-1.01)	0.87 (0.83-0.90)	
	-	117.41	0.89 (0.86-0.94)	0.85 (0.80-0.88)	
	Most deprived	113.43	0.89 (0.86-0.94)	0.85 (0.80-0.89)	

*Negative Binomial model, $ Mixed-effects Negative Binomial Model, ** From multivariate analysis using the likelihood ratio test, ^shows a significance of $p < 0.05$
COPD, Chronic Obstructive Pulmonary Disease; GP, General Practice; OCS, Oral Corticosteroids; BP, Bisphosphonates.

Supplementary Table 6. Bisphosphonates prescribing rates in 2017, stratified by five GP characteristics factors and OCS per 1000 patients in a negative binomial model reporting incidence rate ratio

	Quintile range	Median BP prescription per 1,000 patients	Univariate model IRR (95%CI)*	Multivariate model IRR (95%CI)$	*p*-value**, ^
**Asthma Prevalence (%)**	≤4.84	77.60	Reference	Reference	.5972
	4.85-5.65	108.18	1.29 (1.23-1.36)	1.03 (0.99-1.08)	
	5.66-6.29	122.14	1.44 (1.37-1.52)	1.04 (0.99-1.09)	
	6.30-6.96	131.04	1.55 (1.47-1.63)	1.01 (0.95-1.05)	
	6.97-14.37	134.32	1.63 (1.55-1.71)	1.01 (0.96-1.06)	
**COPD Prevalence (%)**	≤1.14	72.16	Reference	Reference	<.0001
	1.15- 1.60	103.62	1.39 (1.33-1.47)	1.07 (1.02-1.11)	
	1.61-2.04	122.34	1.61 (1.53-1.70)	1.11 (1.05-1.16)	
	2.05-2.64	129.33	1.73 (1.64-1.81)	1.13 (1.07-1.19)	
	2.65-11.25	141.67	1.86 (1.77-1.95)	1.16 (1.09-1.24)	
**OCS per 1000 patients**	≤76.80	53.86	Reference	Reference	<.0001
	76.81-110.54	94.02	1.69 (1.62-1.77)	1.52 (1.45-1.59)	
	110.58-139.54	117.53	2.05 (1.97-2.15)	1.73 (1.65-1.82)	
	139.56-173.95	138.07	2.40 (2.30-2.51)	1.91 (1.81-2.01)	
	≥173.96	168.04	2.99 (2.86-3.13)	2.20 (2.07-2.33)	
**Practice list size**	≤4,127	106.78	Reference	Reference	<.0001
	4,128-6,287	113.85	1.03 (0.98-1.09)	0.99 (0.96-1.03)	
	6,290-8,809	118.50	1.04 (0.99-1.10)	0.97 (0.93-1.01)	
	8,810-12,335	119.72	1.05 (1.01-1.11)	0.94 (0.90-0.97)	
	≥12,336	110.68	0.90 (0.85-0.94)	0.87 (0.84-0.91)	
**QOF score**	≤529.81	97.81	Reference	Reference	.0005
	529.82-545.04	108.15	1.08 (1.03-1.14)	1.07 (1.01-1.10)	
	545.05-552.16	112.34	1.12 (1.07-1.19)	1.06 (1.02-1.10)	
	552.17-557.84	121.22	1.21 (1.15-1.27)	1.06 (1.03-1.11)	
	557.85-559	128.71	1.30 (1.23-1.37)	1.09 (1.04-1.13)	
**% over 65 years old**	≤11	58.16	Reference	Reference	<.0001
	11.01-15.63	99.98	1.59 (1.51-1.66)	1.29 (1.24-1.35)	
	15.64-18.86	120.38	1.91 (1.88-2.00)	1.44 (1.37-1.51)	

	18.87-22.77	135.05	2.13 (2.04-2.24)	1.55 (1.47-1.65)	
	≥22.78	159.22	2.55 (2.43-2.67)	1.77 (1.66-1.89)	
**% patients with a long-term health disease**	≤43.95	80.41	Reference	Reference	.0356
	43.97-49.65	107.05	1.28 (1.22-1.35)	1.02 (0.99-1.06)	
	49.66-53.85	122.56	1.45 (1.38-1.52)	1.03 (0.99-1.08)	
	53.86-58.44	126.80	1.51 (1.43-1.59)	1.03 (0.98-1.08)	
	≥58.45	137.20	1.63 (1.55-1.72)	1.05 (1.01-1.10)	
**IMD score**	Least deprived	124.81	Reference	Reference	<.0001
	-	130.09	1.04 (0.99-1.10)	0.96 (0.92-1.01)	
	-	117.35	0.93 (0.88-0.98)	0.87 (0.84-0.91)	
	-	109.58	0.86 (0.81-0.90)	0.82 (0.78-0.87)	
	Most deprived	91.49	0.77 (0.73-0.81)	0.74 (0.70-0.78)	

*Negative Binomial model, $ Mixed-effects Negative Binomial Model, ** From multivariate analysis using the likelihood ratio test, ^shows a significance of $p < 0.05$
COPD, Chronic Obstructive Pulmonary Disease; GP, General Practice; OCS, Oral Corticosteroids; BP, Bisphosphonates.

In 2016

Supplementary Table 7. Practice summary characteristics in 2016*

	**Median**	**IQR**
**Asthma prevalence (%)**	5.9	5.1 - 6.7
**COPD prevalence (%)**	1.8	1.2 - 2.4
**GP list size**	6,949	4,230 - 10,565
**Patients with long-term health conditions (%)**	53.3	48.2 - 58.4
**Patients over 65 years old (%)**	17.3	12.3 - 21.4
**Quality Outcomes Framework score**	545.9	528.9 - 555.2
**OCS prescribed items per 1,000 patients**	130.5	88.2 - 1167.2
**BP prescribed items per 1,000 patients**	126.5	82.2 - 165.9

*From January to December
COPD, Chronic Obstructive Pulmonary Disease; GP, General Practice; OCS, Oral Corticosteroids; BP, Bisphosphonates.

Supplementary Table 8. Oral corticosteroids prescribing rates in 2016, stratified by five factors in a negative binomial model reporting incidence rate ratio.

	Quintile range	Median OCS prescription per 1,000 patients	Univariate model IRR (95%CI)*	Multivariate model IRR (95%CI)$	*p*-value**, ^
**Asthma Prevalence (%)**	≤4.83	75.42	Reference	Reference	<.0001
	4.84-5.59	114.96	1.42 (1.36-1.48)	1.11 (1.08-1.16)	
	5.60-6.21	132.31	1.61 (1.55-1.68)	1.17 (1.13-1.21)	
	6.22-6.90	151.23	1.81 (1.73-1.89)	1.22 (1.17-1.27)	
	6.91-12.59	163.24	1.97 (1.89-2.06)	1.26 (1.21-1.31)	
**COPD Prevalence (%)**	≤1.13	74.20	Reference	Reference	<.0001
	1.14- 1.58	113.41	1.44 (1.37-1.50)	1.11 (1.08-1.15)	
	1.58-2.01	137.49	1.72 (1.63-1.79)	1.20 (1.15-1.24)	
	2.02-2.60	148.40	1.88 (1.82-1.97)	1.25 (1.20-1.31)	
	2.60-10.78	162.15	2.02 (1.94-2.11)	1.32 (1.25-1.39)	
**GP list size**	≤3,747	110.76	Reference	Reference	<.0001
	3,748-5,808	125.04	1.04 (0.99-1.09)	1.02 (0.98-1.05)	
	5,809-8,172	134.21	1.08 (1.03-1.13)	1.02 (0.99-1.06)	
	8,173-11,514	135.97	1.09 (1.04-1.14)	0.99 (0.96-1.02)	
	≥11,518	128.42	0.97 (0.92-1.01)	0.90 (0.87-0.93)	
**QOF score**	≤522.37	110.48	Reference	Reference	.0140
	522.39-540.63	116.58	1.04 (0.99-1.08)	1.03 (1.01-1.07)	
	540.67-550.00	127.72	1.10 (1.05-1.15)	1.02 (0.99-1.06)	
	550.01-556.39	133.80	1.16 (1.09-1.21)	1.03 (1.01-1.07)	
	556.40-559.00	143.48	1.26 (1.21-1.32)	1.05 (1.01-1.09)	
**% over 65 years old**	≤11	71.67	Reference	Reference	<.0001
	11.01-15.50	111.07	1.45 (1.39-1.52)	1.19 (1.15-1.24)	
	15.51-18.89	134.21	1.70 (1.63-1.77)	1.33 (1.27-1.38)	
	18.90-22.47	144.76	1.84 (1.77-1.92)	1.39 (1.32-1.47)	
	≥22.48	177.19	2.25 (2.16-2.35)	1.62 (1.54-1.71)	
**% patients with a long-**	≤47.00	85.27	Reference	Reference	.0015
	47.01-51.42	114.96	1.28 (1.23-1.34)	1.05 (1.02-1.09)	

**term health disease**	51.43-55.28	134.93	1.46 (1.40-1.53)	1.06 (1.02-1.10)	
	55.28-59.61	143.71	1.59 (1.53-1.66)	1.07 (1.03-1.11)	
	≥59.62	158.45	1.73 (1.65-1.80)	1.07 (1.03-1.11)	
**IMD score**	Least deprived	131.08	Reference	Reference	.0001
	-	140.25	1.06 (0.99-1.08)	0.93 (0.89-0.96)	
	-	128.13	0.96 (0.92-1.01)	0.87 (0.84-0.91)	
	-	120.57	0.90 (0.86-0.95)	0.85 (0.81-0.89)	
	Most deprived	120.61	0.92 (0.88-0.97)	0.86 (0.82-0.90)	

*Negative Binomial model, $ Mixed-effects Negative Binomial Model, ** From multivariate analysis using the likelihood ratio test, ^shows a significance of $p < 0.05$

COPD, Chronic Obstructive Pulmonary Disease; GP, General Practice; OCS, Oral Corticosteroids; BP, Bisphosphonates.

Supplementary Table 9. Bisphosphonates prescribing rates in 2016, stratified by five factors and OCS per 1,000 patients in a negative binomial model reporting incidence rate ratio.

	Quintile range	Median BP prescription per 1,000 patients	Univariate model IRR (95%CI)*	Multivariate model IRR (95%CI)$	*p*-value**, ^
**Asthma Prevalence (%)**	≤4.83	84.23	Reference	Reference	.5919
	4.84-5.59	114.71	1.28 (1.22-1.35)	1.02 (0.98-1.06)	
	5.60-6.21	129.58	1.42 (1.35-1.50)	1.03 (0.98-1.08)	
	6.22-6.90	138.70	1.54 (1.47-1.62)	0.99 (0.94-1.04)	
	6.91-12.59	145.62	1.61 (1.54-1.70)	1.01 (0.96-1.06)	
**COPD Prevalence (%)**	≤1.13	76.45	Reference	Reference	<.0001
	1.14- 1.58	111.89	1.39 (1.33-1.47)	1.05 (1.01-1.10)	
	1.58-2.01	132.02	1.60 (1.52-1.68)	1.08 (1.04-1.14)	
	2.02-2.60	138.61	1.72 (1.63-1.80)	1.10 (1.05-1.16)	
	2.60-10.78	151.72	1.84 (1.75-1.93)	1.13 (1.06-1.20)	
**OCS per 1000 patients**	≤78.23	59.84	Reference	Reference	<.0001
	78.26-112.50	99.81	1.62 (1.55-1.69)	1.46 (1.40-1.52)	
	112.51-141.98	124.55	1.99 (1.91-2.08)	1.65 (1.57-1.74)	
	141.98-176.62	147.60	2.31 (2.21-2.42)	1.81 (1.72-1.90)	
	≥176.63	182.25	2.90 (2.77-3.03)	2.10 (1.98-2.23)	
**GP list size**	≤3,747	113.97	Reference	Reference	<.0001
	3,748-5,808	119.23	1.01 (0.96-1.07)	0.97 (0.93-1.01)	
	5,809-8,172	126.60	1.06 (1.01-1.11)	0.97 (0.93-1.01)	
	8,173-11,514	129.41	1.07 (1.02-1.13)	0.94 (0.90-0.98)	
	≥11,518	120.37	0.94 (0.89-0.99)	0.88 (0.84-0.92)	
**QOF score**	≤522.37	107.32	Reference	Reference	.0011
	522.39-540.63	110.28	1.02 (0.96-1.07)	1.01 (0.96-1.04)	
	540.67-550.00	124.38	1.13 (1.07-1.19)	1.04 (1.01-1.08)	
	550.01-556.39	128.91	1.17 (1.11-1.22)	1.04 (1.01-1.08)	
	556.40-559.00	137.60	1.26 (1.20-1.32)	1.07 (1.02-1.11)	
**% over 65 years old**	≤11	62.86	Reference	Reference	<.0001
	11.01-15.50	107.04	1.61 (1.54-1.69)	1.32 (1.26-1.38)	
	15.51-18.89	126.89	1.92 (1.83-2.01)	1.46 (1.38-1.53)	

	18.90-22.47	143.74	2.14 (2.04-2.24)	1.56 (1.48-1.65)	
	≥22.48	170.81	2.54 (2.43-2.67)	1.75 (1.64-1.87)	
**% patients with a long-term health disease**	≤47.00	84.69	Reference	Reference	.0089
	47.01-51.42	115.65	1.29 (1.22-1.35)	1.05 (1.02-1.09)	
	51.43-55.28	126.42	1.40 (1.33-1.47)	1.04 (0.99-1.08)	
	55.28-59.61	136.97	1.54 (1.46-1.62)	1.05 (1.02-1.09)	
	≥59.62	152.16	1.69 (1.60-1.77)	1.07 (1.02-1.12)	
**IMD score**	Least deprived	132.87	Reference	Reference	<.0001
	-	138.44	1.03 (0.98-1.07)	0.95 (0.92-0.99)	
	-	125.58	0.93 (0.88-0.98)	0.87 (0.84-0.92)	
	-	117.26	0.86 (0.82-0.91)	0.82 (0.78-0.86)	
	Most deprived	100.18	0.77 (0.74-0.81)	0.73 (0.69-0.77)	

*Negative Binomial model, $ Mixed-effects Negative Binomial Model, ** From multivariate analysis using the likelihood ratio test, ^shows a significance of $p < 0.05$
COPD, Chronic Obstructive Pulmonary Disease; GP, General Practice; OCS, Oral Corticosteroids; BP, Bisphosphonates.

In 2015

Supplementary Table 10. Practice summary characteristics in 2015*

	Median	IQR
**Asthma prevalence (%)**	6.0	5.1 - 6.8
**COPD prevalence (%)**	1.7	1.2 - 2.4
**GP list size**	7,044	4,380 - 10,538
**Patients with long-term health conditions (%)**	54.2	49.8 - 59.3
**Patients over 65 years old (%)**	17.2	12.3 - 21.23
**Quality Outcomes Framework score**	543.3	524.3 - 553.6
**OCS prescribed items per 1,000 patients**	128.8	92.5 - 167.6
**BP prescribed items per 1,000 patients**	134.8	91.9 - 186.5

*From January to December
COPD, Chronic Obstructive Pulmonary Disease; GP, General Practice; OCS, Oral Corticosteroids; BP, Bisphosphonates.

Supplementary Table 11. Oral corticosteroids prescribing rates in 2015, stratified by five factors in a negative binomial model reporting incidence rate ratio.

	Quintile range	Median OCS prescription per 1,000 patients	Univariate model IRR (95%CI)*	Multivariate model IRR (95%CI)$	*p*-value**, ^
**Asthma Prevalence (%)**	≤4.90	75.58	Reference	Reference	<.0001
	4.91-5.66	113.06	1.39 (1.33-1.46)	1.12 (1.08-1.16)	
	5.67-6.29	129.62	1.59 (1.52-1.66)	1.20 (1.12-1.21)	
	6.30-7.00	145.39	1.74 (1.67-1.83)	1.24 (1.15-1.25)	
	7.01-12.45	158.53	1.92 (1.83-2.00)	1.33 (1.21-1.32)	
**COPD Prevalence (%)**	≤1.10	73.57	Reference	Reference	<.0001
	1.11-1.55	111.50	1.44 (1.37-1.50)	1.13 (1.09-1.17)	
	1.56-1.97	135.04	1.72 (1.64-1.80)	1.20 (1.14-1.25)	
	1.98-2.55	145.39	1.85 (1.77-1.93)	1.24 (1.19-1.30)	
	2.56-9.01	158.80	2.01 (1.92-2.10)	1.33 (1.26-1.40)	
**GP list size**	≤3,540	109.05	Reference	Reference	<.0001
	3,543-5,587	121.06	1.04 (0.99-1.09)	1.01 (0.97-1.05)	
	5,589-7,942	131.96	1.10 (1.05-1.15)	1.02 (0.98-1.06)	
	7,943-11,221	131.74	1.09 (1.04-1.15)	0.99 (0.95-1.02)	
	≥11,230	126.17	0.98 (0.93-1.03)	0.90 (0.86-0.93)	
**QOF score**	≤516.27	107.36	Reference	Reference	.0061
	516.28-536.62	119.87	1.08 (1.03-1.13)	1.02 (0.98-1.06)	
	536.63-547.52	125.68	1.13 (1.07-1.18)	1.04 (1.01-1.08)	
	547.53-555.09	128.82	1.16 (1.10-1.21)	1.03 (0.99-1.07)	
	555.10-559.00	142.72	1.27 (1.21-1.33)	1.06 (1.02-1.10)	
**% over 65 years old**	≤10.93	70.62	Reference	Reference	<.0001
	10.93-15.43	108.05	1.47 (1.41-1.54)	1.20 (1.15-1.25)	
	15.44-18.76	131.07	1.71 (1.63-1.78)	1.32 (1.25-1.38)	
	18.77-22.28	142.67	1.87 (1.79-1.95)	1.42 (1.35-1.49)	
	≥22.29	172.66	2.27 (2.18-2.37)	1.62 (1.53-1.71)	
**% patients with a long-term health disease**	≤47.40	83.42	Reference	Reference	.0003
	47.42-52.21	118.13	1.33 (1.27-1.39)	1.07 (1.03-1.11)	
	52.22-56.18	129.89	1.45 (1.39-1.52)	1.07 (1.03-1.11)	

	56.19-60.65	140.17	1.58 (1.51-1.65)	1.07 (1.03-1.12)	
	≥60.66	155.16	1.73 (1.65-1.81)	1.09 (1.05-1.13)	
**IMD score**	Least deprived	129.43	Reference	Reference	<.0001
	-	138.41	1.04 (0.99-1.10)	0.93 (0.90-0.97)	
	-	125.87	0.97 (0.93-1.02)	0.87 (0.84-0.91)	
	-	115.76	0.89 (0.85-0.94)	0.84 (0.80-0.88)	
	Most deprived	114.37	0.90 (0.86-0.95)	0.84 (0.80-0.88)	

*Negative Binomial model, $ Mixed-effects Negative Binomial Model, ** From multivariate analysis using the likelihood ratio test, ^shows a significance of $p < 0.05$
COPD, Chronic Obstructive Pulmonary Disease; GP, General Practice; OCS, Oral Corticosteroids; BP, Bisphosphonates.

Supplementary Table 12. Bisphosphonates prescribing rates in 2015, stratified by five factors and OCS per 1,000 patients in a negative binomial model reporting incidence rate ratio.

	Quintile range	Median BP prescription per 1,000 patients	Univariate model IRR (95%CI)*	Multivariate model IRR (95%CI)$	*p*-value**, ^
**Asthma Prevalence (%)**	≤4.90	88.65	Reference	Reference	.2614
	4.91-5.66	124.46	1.30 (1.24-1.37)	1.03 (0.98-1.07)	
	5.67-6.29	137.73	1.44 (1.37-1.52)	1.01 (0.96-1.05)	
	6.30-7.00	146.46	1.55 (1.47-1.63)	1.01 (0.95-1.05)	
	7.01-12.45	152.55	1.60 (1.52-1.68)	0.99 (0.94-1.04)	
**COPD Prevalence (%)**	≤1.10	81.56	Reference	Reference	<.0001
	1.11-1.55	118.06	1.40 (1.33-1.47)	1.06 (1.02-1.12)	
	1.56-1.97	141.46	1.63 (1.55-1.71)	1.10 (1.05-1.15)	
	1.98-2.55	148.03	1.72 (1.64-1.81)	1.12 (1.07-1.18)	
	2.56-9.01	158.55	1.84 (1.75-1.94)	1.15 (1.08-1.22)	
**OCS per 1000 patients**	≤77.31	61.16	Reference	Reference	<.0001
	77.33-110.27	106.01	1.68 (1.61-1.76)	1.46 (1.40-1.53)	
	110.28-139.07	130.31	2.04 (1.95-2.14)	1.63 (1.55-1.71)	
	139.11-174.28	154.11	2.35 (2.25-2.46)	1.76 (1.66-1.85)	
	≥174.36	191.96	2.98 (2.85-3.11)	2.06 (1.94-2.18)	
**GP list size**	≤3,540	121.02	Reference	Reference	<.0001
	3,543-5,587	124.93	1.02 (0.97-1.07)	0.97 (0.93-1.01)	
	5,589-7,942	133.67	1.08 (1.02-1.13)	0.96 (0.92-0.99)	
	7,943-11,221	136.78	1.08 (1.03-1.14)	0.95 (0.91-0.99)	
	≥11,230	127.49	0.96 (0.91-1.01)	0.86 (0.82-0.90)	
**QOF score**	≤516.27	113.37	Reference	Reference	<.0001
	516.28-536.62	121.52	1.07 (1.02-1.12)	1.05 (1.01-1.08)	
	536.63-547.52	128.38	1.14 (1.08-1.20)	1.06 (1.02-1.11)	
	547.53-555.09	133.59	1.18 (1.12-1.24)	1.07 (1.03-1.11)	
	555.10-559.00	146.91	1.29 (1.23-1.36)	1.09 (1.05-1.14)	
**% over 65 years old**	≤10.93	65.39	Reference	Reference	<.0001
	10.93-15.43	113.04	1.65 (1.57-1.72)	1.32 (1.26-1.38)	
	15.44-18.76	136.50	1.97 (1.88-2.06)	1.46 (1.37-1.54)	

	18.77-22.28	154.11	2.17 (2.07-2.28)	1.54 (1.46-1.63)	
	≥22.29	184.27	2.62 (2.50-2.75)	1.73 (1.62-1.85)	
**% patients with a long-term health disease**	≤47.40	88.20	Reference	Reference	<.0001
	47.42-52.21	122.85	1.32 (1.25-1.39)	1.05 (1.01-1.09)	
	52.22-56.18	132.23	1.42 (1.35-1.50)	1.05 (1.01-1.10)	
	56.19-60.65	147.75	1.57 (1.49-1.65)	1.08 (1.04-1.13)	
	≥60.66	156.66	1.68 (1.60-1.77)	1.10 (1.05-1.15)	
**IMD score**	Least deprived	140.46	Reference	Reference	<.0001
	-	149.19	1.03 (0.98-1.08)	0.93 (0.90-0.97)	
	-	131.13	0.92 (0.88-0.97)	0.86 (0.82-0.90)	
	-	121.36	0.85 (0.80-0.89)	0.80 (0.75-0.84)	
	Most deprived	103.66	0.76 (0.72-0.80)	0.71 (0.67-0.75)	

*Negative Binomial model, $ Mixed-effects Negative Binomial Model, ** From multivariate analysis using the likelihood ratio test, ^shows a significance of $p < 0.05$
COPD, Chronic Obstructive Pulmonary Disease; GP, General Practice; OCS, Oral Corticosteroids; BP, Bisphosphonates.
